# Supplementary material for: Hot spot prediction in protein-protein interactions by an ensemble system
Source: BMC Syst Biol. 2018 Dec 31;12(Suppl 9):132. doi: 10.1186/s12918-018-0665-8 (PMC6311905; doi:10.1186/s12918-018-0665-8)
Supplement: Supplementary file 2 — All relevant MATLAB code. A simple Matlab implement of our predictor. (ZIP 19,742 kb) [file 12918_2018_665_MOESM2_ESM.zip › Additional file 2/Read ASA/ASA Property/SKEMPI/2J0TD.txt]

 NetSurfP - Protein Surface Accessibility and Secondary Structure Predictions

  

|  |  |  |
| --- | --- | --- |
|  |  | NetSurfP - Protein Surface Accessibility and Secondary Structure Predictions  Technical University of Denmark |

  


---

```
# For publication of results, please cite:
# A generic method for assignment of reliability scores applied to solvent accessibility predictions.
# Bent Petersen, Thomas Nordahl Petersen, Pernille Andersen, Morten Nielsen and Claus Lundegaard
# BMC Structural Biology 2009, 9:51 doi:10.1186/1472-6807-9-51
#
# Column 1: Class assignment - B for buried or E for Exposed - Threshold: 25% exposure, but not based on RSA
# Column 2: Amino acid
# Column 3: Sequence name
# Column 4: Amino acid number
# Column 5: Relative Surface Accessibility - RSA
# Column 6: Absolute Surface Accessibility
# Column 7: Z-fit score for RSA prediction
# Column 8: Probability for Alpha-Helix
# Column 9: Probability for Beta-strand
# Column 10: Probability for Coil
E C  2J0TD                  1    0.474  66.536  -2.447   0.003   0.003   0.994
E T  2J0TD                  2    0.624  86.604   0.000   0.005   0.262   0.733
B C  2J0TD                  3    0.183  25.763  -2.284   0.004   0.197   0.799
E V  2J0TD                  4    0.530  81.461   0.184   0.004   0.085   0.910
E P  2J0TD                  5    0.511  72.511  -1.274   0.018   0.047   0.935
E P  2J0TD                  6    0.655  92.973  -0.098   0.018   0.047   0.935
E H  2J0TD                  7    0.286  51.987   0.115   0.018   0.019   0.964
E P  2J0TD                  8    0.335  47.508  -0.520   0.058   0.017   0.925
E Q  2J0TD                  9    0.433  77.423   0.295   0.113   0.043   0.844
E T  2J0TD                 10    0.515  71.403  -0.024   0.113   0.087   0.800
B A  2J0TD                 11    0.271  29.820   0.417   0.118   0.150   0.732
B F  2J0TD                 12    0.154  30.908   0.235   0.125   0.227   0.648
B C  2J0TD                 13    0.058   8.185   0.761   0.066   0.296   0.638
E N  2J0TD                 14    0.445  65.163   0.485   0.064   0.216   0.721
B S  2J0TD                 15    0.081   9.435   0.368   0.020   0.205   0.775
E D  2J0TD                 16    0.313  45.074   0.508   0.021   0.451   0.528
B L  2J0TD                 17    0.062  11.444   0.966   0.018   0.846   0.136
B V  2J0TD                 18    0.040   6.148  -0.147   0.006   0.962   0.032
B I  2J0TD                 19    0.036   6.568   0.390   0.001   0.959   0.040
B R  2J0TD                 20    0.150  34.304   1.285   0.001   0.959   0.040
B A  2J0TD                 21    0.026   2.898   0.347   0.001   0.959   0.040
E K  2J0TD                 22    0.285  58.686   2.093   0.001   0.959   0.040
B F  2J0TD                 23    0.061  12.162   0.344   0.001   0.959   0.040
B V  2J0TD                 24    0.131  20.181  -0.134   0.002   0.816   0.182
E G  2J0TD                 25    0.354  27.860  -0.837   0.004   0.514   0.481
E T  2J0TD                 26    0.453  62.831   0.602   0.005   0.262   0.733
E P  2J0TD                 27    0.633  89.865  -0.222   0.020   0.205   0.775
E E  2J0TD                 28    0.571  99.719  -0.334   0.020   0.205   0.775
B V  2J0TD                 29    0.209  32.077  -1.540   0.019   0.141   0.840
E N  2J0TD                 30    0.548  80.242  -0.865   0.018   0.047   0.935
E Q  2J0TD                 31    0.840 150.006  -0.083   0.018   0.047   0.935
E T  2J0TD                 32    0.388  53.760  -1.122   0.018   0.047   0.935
E T  2J0TD                 33    0.567  78.615  -0.903   0.018   0.088   0.893
B L  2J0TD                 34    0.263  48.229  -1.433   0.021   0.279   0.699
E Y  2J0TD                 35    0.380  81.163  -0.809   0.004   0.514   0.481
E Q  2J0TD                 36    0.325  58.116  -0.344   0.003   0.718   0.279
E R  2J0TD                 37    0.404  92.630  -0.128   0.002   0.816   0.182
B Y  2J0TD                 38    0.111  23.785   0.468   0.001   0.900   0.099
E E  2J0TD                 39    0.444  77.637   0.988   0.001   0.959   0.040
B I  2J0TD                 40    0.110  20.257   0.115   0.001   0.959   0.040
E K  2J0TD                 41    0.465  95.712   1.045   0.001   0.959   0.040
B M  2J0TD                 42    0.207  41.361   1.008   0.001   0.959   0.040
E T  2J0TD                 43    0.412  57.144   1.320   0.001   0.959   0.040
E K  2J0TD                 44    0.338  69.444   0.482   0.001   0.959   0.040
B M  2J0TD                 45    0.165  33.037  -0.381   0.011   0.918   0.071
B Y  2J0TD                 46    0.140  29.875   0.102   0.003   0.718   0.279
E K  2J0TD                 47    0.372  76.418  -0.396   0.004   0.420   0.576
B G  2J0TD                 48    0.251  19.769  -1.806   0.018   0.088   0.893
E F  2J0TD                 49    0.273  54.831  -1.267   0.018   0.047   0.935
E Q  2J0TD                 50    0.674 120.376  -0.320   0.053   0.043   0.903
E A  2J0TD                 51    0.518  57.128  -0.809   0.052   0.084   0.864
B L  2J0TD                 52    0.122  22.302  -0.620   0.052   0.084   0.864
E G  2J0TD                 53    0.498  39.200  -0.006   0.052   0.084   0.864
E D  2J0TD                 54    0.589  84.933  -0.170   0.052   0.084   0.864
B A  2J0TD                 55    0.282  31.043  -1.446   0.018   0.088   0.893
E A  2J0TD                 56    0.451  49.711  -0.817   0.018   0.088   0.893
E D  2J0TD                 57    0.461  66.488   0.437   0.004   0.138   0.858
B I  2J0TD                 58    0.079  14.652   0.203   0.004   0.616   0.381
B R  2J0TD                 59    0.142  32.449   0.221   0.002   0.816   0.182
B F  2J0TD                 60    0.180  36.086   0.361   0.001   0.900   0.099
B V  2J0TD                 61    0.044   6.701   0.273   0.001   0.900   0.099
B Y  2J0TD                 62    0.152  32.547   0.478   0.002   0.816   0.182
B T  2J0TD                 63    0.077  10.652  -0.269   0.005   0.336   0.660
B P  2J0TD                 64    0.209  29.629  -0.111   0.018   0.088   0.893
E A  2J0TD                 65    0.302  33.269  -1.273   0.018   0.047   0.935
E M  2J0TD                 66    0.531 106.293  -1.638   0.018   0.047   0.935
E E  2J0TD                 67    0.562  98.129  -1.849   0.018   0.019   0.964
E S  2J0TD                 68    0.526  61.600  -1.578   0.053   0.043   0.903
B V  2J0TD                 69    0.240  36.934  -1.542   0.052   0.084   0.864
B C  2J0TD                 70    0.051   7.188  -0.506   0.019   0.141   0.840
B G  2J0TD                 71    0.208  16.346  -1.884   0.021   0.279   0.699
B Y  2J0TD                 72    0.222  47.441  -0.216   0.021   0.451   0.528
E F  2J0TD                 73    0.448  89.954   0.614   0.023   0.655   0.322
B H  2J0TD                 74    0.179  32.615   0.519   0.022   0.552   0.426
E R  2J0TD                 75    0.552 126.477   0.141   0.022   0.359   0.619
E S  2J0TD                 76    0.374  43.809  -0.618   0.020   0.205   0.775
E H  2J0TD                 77    0.437  79.527  -0.989   0.018   0.088   0.893
E N  2J0TD                 78    0.665  97.283  -0.817   0.018   0.047   0.935
E R  2J0TD                 79    0.508 116.401   0.317   0.018   0.088   0.893
E S  2J0TD                 80    0.413  48.427  -0.386   0.004   0.138   0.858
E E  2J0TD                 81    0.370  64.604   0.975   0.004   0.514   0.481
B E  2J0TD                 82    0.176  30.695   0.740   0.002   0.816   0.182
B F  2J0TD                 83    0.050   9.935   1.258   0.001   0.900   0.099
B L  2J0TD                 84    0.029   5.383   0.677   0.001   0.959   0.040
B I  2J0TD                 85    0.029   5.328   0.119   0.001   0.959   0.040
B A  2J0TD                 86    0.025   2.766  -0.369   0.001   0.900   0.099
B G  2J0TD                 87    0.061   4.816  -0.995   0.002   0.816   0.182
E K  2J0TD                 88    0.313  64.364   0.434   0.001   0.900   0.099
B L  2J0TD                 89    0.083  15.142  -0.668   0.002   0.816   0.182
E Q  2J0TD                 90    0.495  88.371  -0.272   0.004   0.420   0.576
E D  2J0TD                 91    0.676  97.354  -0.999   0.005   0.015   0.979
E G  2J0TD                 92    0.373  29.363  -1.217   0.005   0.015   0.979
E L  2J0TD                 93    0.404  73.936   1.328   0.003   0.718   0.279
B L  2J0TD                 94    0.063  11.554   0.651   0.001   0.959   0.040
B H  2J0TD                 95    0.172  31.250   1.117   0.001   0.959   0.040
B I  2J0TD                 96    0.059  11.007   0.437   0.001   0.959   0.040
B T  2J0TD                 97    0.089  12.372  -0.795   0.001   0.900   0.099
B T  2J0TD                 98    0.205  28.447  -0.377   0.002   0.816   0.182
B C  2J0TD                 99    0.047   6.655  -0.235   0.021   0.451   0.528
B S  2J0TD                100    0.282  33.015  -0.636   0.022   0.359   0.619
B F  2J0TD                101    0.183  36.828  -0.554   0.022   0.552   0.426
E V  2J0TD                102    0.324  49.783   0.291   0.022   0.552   0.426
E A  2J0TD                103    0.327  36.013   0.971   0.022   0.359   0.619
E P  2J0TD                104    0.405  57.484  -0.020   0.021   0.279   0.699
B W  2J0TD                105    0.158  38.023   0.042   0.020   0.205   0.775
E N  2J0TD                106    0.556  81.384   0.148   0.056   0.142   0.802
E S  2J0TD                107    0.572  67.038   0.831   0.056   0.142   0.802
B L  2J0TD                108    0.101  18.511   0.572   0.113   0.043   0.844
E S  2J0TD                109    0.396  46.423   1.180   0.053   0.043   0.903
E L  2J0TD                110    0.508  92.978   0.865   0.858   0.002   0.139
E A  2J0TD                111    0.531  58.494   0.581   0.938   0.007   0.055
B Q  2J0TD                112    0.209  37.417   0.007   0.879   0.010   0.111
E R  2J0TD                113    0.408  93.409   0.488   0.879   0.010   0.111
E R  2J0TD                114    0.482 110.286   0.945   0.717   0.014   0.269
E G  2J0TD                115    0.310  24.421  -1.053   0.600   0.003   0.397
B F  2J0TD                116    0.092  18.484   0.265   0.694   0.003   0.303
E T  2J0TD                117    0.527  73.137   1.233   0.879   0.010   0.111
E K  2J0TD                118    0.516 106.162   0.398   0.879   0.010   0.111
E T  2J0TD                119    0.258  35.785   1.195   0.879   0.010   0.111
B Y  2J0TD                120    0.232  49.643  -0.195   0.879   0.010   0.111
E T  2J0TD                121    0.620  85.994   0.689   0.802   0.014   0.185
E V  2J0TD                122    0.582  89.377   0.742   0.430   0.016   0.555
E G  2J0TD                123    0.613  48.275  -0.405   0.181   0.016   0.803
E C  2J0TD                124    0.540  75.802  -1.197   0.003   0.003   0.994

Explain the output.  Go back.
```

---
